# Glycyrrhizin Attenuates Hypoxic-Ischemic Brain Damage by Inhibiting Ferroptosis and Neuroinflammation in Neonatal Rats via the HMGB1/GPX4 Pathway

**DOI:** 10.1155/2022/8438528

**Published:** 2022-04-07

**Authors:** Kaiyi Zhu, Xing Zhu, Shiqi Liu, Jie Yu, Songwei Wu, Mingyan Hei

**Affiliations:** ^1^Department of Neonatology, Neonatal Center, Beijing Children's Hospital, Capital Medical University, National Center for Children's Health, Beijing 100045, China; ^2^Key Laboratory of Major Diseases in Children, Ministry of Education, Beijing 100045, China

## Abstract

With unknown etiology and limited treatment options, neonatal hypoxic-ischemic brain damage (HIBD) remains a major cause of mortality in newborns. Ferroptosis, a recently discovered type of cell death triggered by lipid peroxidation, is closely associated with HIBD. High-mobility group box 1 (HMGB1), a molecule associated with inflammation damage, can induce neuronal death in HIBD. However, it remains unknown whether HMGB1 contributes to neuronal ferroptosis in patients with HIBD. Herein, glycyrrhizin (GL), an HMGB1 inhibitor, was used to investigate the relationship between ferroptosis and HMGB1. RAS-selective lethal 3(RSL3), a ferroptosis agonist, was administered to further confirm the changes in the signaling pathway between HMGB1 and ferroptosis. Western blot analysis revealed that GL markedly suppressed the expression of HMGB1 and increased the level of GPX4 in the context of HIBD. We observed changes in neuronal ultrastructure via transmission electron microscopy to further confirm the occurrence of ferroptosis. Real-time PCR indicated that GL inhibited the expression of ferroptosis-related genes and inflammatory factors. Immunofluorescence and immunohistochemistry staining confirmed GL inhibition of neuronal damage and ferroptosis in HIBD associated with GPX4 and ROS. GL not only inhibited ferroptosis induced by RSL3 and oxygen-glucose deprivation in vitro but also inhibited ferroptosis induced by HIBD in vivo. More importantly, GL may improve oxidative stress imbalance and mitochondrial damage, alleviate the downstream production of inflammatory factors, and ultimately reduce ferroptosis and damage to cortical neurons following HIBD via the HMGB1/GPX4 pathway. In conclusion, we showed for the first time that GL could suppress the occurrence of neuronal ferroptosis and reduce neuronal loss in HIBD via the HMGB1/GPX4 pathway. These findings highlight the potential of HMGB1 signaling antagonists to treat neuronal damage by suppressing ferroptosis, provide new and unique insights into GL as a neuroprotective agent, and suggest new prevention and treatment strategies for HIBD.

## 1. Introduction

Neonatal hypoxic-ischemic brain damage (HIBD) remains a major cause of mortality in newborns during the perinatal period [1, 2]. Survivors exhibit an increased incidence of severe and permanent neurological sequelae, including cerebral palsy, cognitive impairment, learning impairment, developmental delay, and epilepsy; these sequelae compromise the quality of life of HIBD children and impose an immense economic and psychological burden on families and society [1–3]. To date, hypothermia is the only recognized therapy for newborns with moderate to severe HIBD; however, this treatment has limited efficacy [2, 4]. The exact pathogenesis of HIBD is a complex process that is not fully understood; it includes oxidative stress, mitochondrial damage, and inflammation, eventually leading to progressive neuronal death [4, 5].

Ferroptosis is a recently discovered, caspase-independent type of cell death that is triggered by lipid peroxidation and is genetically distinct from other types of cell death [6, 7]. The occurrence of ferroptosis is independent of the mediators of apoptosis (caspase-3), necroptosis (RIP1 or RIP3), and pyroptosis (caspase-1 or caspase-11) [8]. Cell death by ferroptosis is caused by factors such as iron toxicity, glutathione (GSH) depletion, glutathione peroxidase 4 (GPX4) impairment, formation of excessive lipid peroxides and reactive oxygen species (ROS), and mitochondrial damage [9, 10]. The induction of ferroptosis through GSH deficiency is related to decreased GPX4 activity, resulting in the accumulation of membrane lipid peroxides and lethal ROS [11–13]. Ferroptosis is distinguished morphologically by mitochondrial shrinkage and is regulated by a number of genes, including ATP synthase F0 complex subunit C3 (ATP5G3), prostaglandin-endoperoxide synthase 2 (PTGS2), citrate synthase (CS), iron response element binding protein 2 (IREB2), and ribosomal protein L8 (RPL8) [8, 14, 15]. In addition, ferroptosis can potently induce inflammation through the release of proinflammatory molecules such as tumor necrosis factor- (TNF-) *α*, interleukin- (IL-) 1*β*, and IL-6 [15, 16]. Ferroptosis can be triggered by specific inducers, including erastin and RAS-selective lethal 3 (RSL3), and can be specifically inhibited by ferrostatin-1 and liproxstatin-1 [8, 17]. At present, there is accumulating evidence that ferroptosis plays a very important role in central nervous system (CNS) diseases, including intracerebral hemorrhage, traumatic brain injury and even HIBD, and inhibiting ferroptosis can prevent neuronal death in some CNS diseases [7, 18–20]. However, the clinical development of ferroptosis inhibitors for the treatment of brain diseases is limited because they have difficulty crossing the blood–brain barrier [18, 21]. Thus, there is a need to elucidate the underlying mechanisms of ferroptosis in HIBD and investigate potential antiferroptosis drugs to prevent and treat HIBD.

Glycyrrhizin (GL) is a natural glycosyl triterpenoid product and is recognized as an inhibitor of high-mobility group box 1 (HMGB1) [22]. HMGB1 is a ubiquitous transcription factor that is involved in the maintenance of nucleosome structure, chromatin remodeling, and the regulation of DNA recombination and repair [23–25]. Numerous studies have shown that HMGB1 is released from damaged cells and exhibits cytokine activity that contributes to the pathogenesis of numerous CNS diseases, including HIBD [24]. Our early studies showed that HMGB1 was upregulated in both the cerebral cortex and serum in HIBD and that GL decreased the ipsilateral infarct size and the degree of cerebral edema [26]. Recent studies have shown that ferroptosis mediates HMGB1 release and subsequent inflammation in an acute pancreatitis model [6]. It is still unclear, however, whether the inhibition of HMGB1 can ameliorate inflammation and brain damage in HIBD by inhibiting ferroptosis.

In the present study, GL, the main extract from the root of *Glycyrrhiza*, was used to investigate whether HMGB1 inhibition alleviates brain damage related to ferroptosis in vivo and in vitro. In addition, RSL3, an agonist of ferroptosis, was administered to further confirm the relationship between GL and ferroptosis and the corresponding changes in signaling pathways. The aim of this study may reveal a novel function of GL in ferroptosis in a rat model and identify a possible therapeutic strategy that could be translated from bench to bedside for HIBD and possibly even other ferroptosis-related diseases.

## 2. Materials and Methods

### 2.1. HIBD Animal Model and Drug Administration

Male and female neonatal Sprague–Dawley rats on postpartum day 7 (P7) were provided by SPF Biotechnology (Beijing, China). A neonatal rat HIBD model was established based on a previously described method [27]. In short, each animal was anesthetized with isoflurane (4% for induction, 2% for maintenance), the skin was incised, and the left common carotid artery was exposed. This artery was ligated with a 5-0 suture and cut, and the skin was sutured closed. Next, the pups recovered for 1 h with their mother. Subsequently, the pups were placed in a hypoxia chamber (8% O_2_+92% N_2_ mixture) for 2 h. After 2 h of hypoxia, the animals were placed back with their dam. The rat pups were randomly divided into 4 groups: (1) in the control (Con) group, the common carotid artery was merely exposed and separated from the left common carotid artery; (2) in the hypoxia-ischemia (HI) group, the pups underwent cerebral HIBD; (3) in the HI + glycyrrhizin (HI + GL) group, the pups received GL (20 mg/kg, Selleck, USA) injections intraperitoneally at 0 h, 24 h, and 48 h after HIBD; and (4) in the HI + GL+ RSL3 group, the rats in the HI + GL group received RSL3 (5 mg/kg, Selleck, USA) treatment at the same time. The rats were euthanized at P10 for subsequent analysis.

### 2.2. Primary Cortical Neuron Culture and Treatment

Rat pups at P1 were decapitated, the brains were collected, and the meninges were removed. The brain tissue was fragmented, digested with trypsin, and filtered with a 50 mm sterile nylon filter. Cortical neurons for differentiation and treatment were cultured in Dulbecco's Modified Eagle's Medium (DMEM; HyClone, Logan, Utah, USA) supplemented with 100 U/mL penicillin and 100 mg/mL streptomycin and 10% fetal bovine serum (FBS, HyClone, USA). To establish an oxygen-glucose deprivation (OGD) model, the cells were cultured in a glucose-free Earle's balanced salt solution with 100 U/mL penicillin and 100 mg/mL streptomycin and incubated under an atmosphere of 5% CO_2_ and 1% O_2_ for 3 h at 37°C in a tri-gas incubator (HERA cell VIOS 160i, Thermo, USA).

To detect the effect of GL on OGD-treated cells, the cells were divided into the following groups: (1) Con group: the cells were cultured with untreated medium in a normal incubator, (2) GL group: the conditions were the same as in the Con group except for the addition of GL (55 *μ*M), (3) OGD group: the cells were subjected to OGD, and (4) OGD + GL group: GL was added to the wells 2 h before the cells were incubated under OGD conditions. To investigate the function of GL on RSL3-induced ferroptosis, the cells were divided into four groups: (1) the Con group, (2) the GL group, (3) the RSL3 group, in which the cells were treated with RSL3 (0.1 *μ*M) for 12 h under normal conditions, and (4) the RSL3 + GL group, in which the cells were treated with GL for 2 h and subsequently cultured in medium with RSL3. To further verify the relationship between GL and ferroptosis, the cells were grouped as follows: (1) the Con group, (2) the OGD group, (3) the OGD + GL group, and (4) the OGD + RSL3 + GL group, in which the cells were cultured for 9 h with RSL3 and GL and then subjected to OGD treatment. In the Con and OGD group, an equal volume of DMSO was administered.

For the in vitro procedure, RSL3 was dissolved in 1% dimethyl sulfoxide (DMSO) at a concentration of 100 *μ*M for storage and further diluted in medium to the required concentration of 0.1 *μ*M; GL was dissolved in 1% dimethyl DMSO at a concentration of 55 mm for storage and further diluted in medium to the required concentration of 55 *μ*M.

### 2.3. Cell Viability Assay

An MTT assay was used to evaluate cell viability in vitro. The cell density of the groups was adjusted prior to the cell viability assay, and cells were seeded in 24-well plates at a density of 2 × 10^4^ cells/well. The cells were cultivated for 4 h in medium containing 3-(4,5-dimethylthiazol-2-yl)-2,5-diphenyltetrazolium bromide (0.5 mg/mL) at the indicated time points. The supernatant was aspirated, the cells were suspended in DMSO, and the optical density (OD) was quantified at 490 nm. The cell viability was calculated after quantifying the absorbances in the MTT assay using the following formula: cell viability (%) = OD of treated group/OD of control group × 100.

### 2.4. Oxidative Stress Analysis

The activity of various oxidative stress indicators, including malondialdehyde (MDA), catalase (CAT), GSH, superoxide dismutase (SOD), Mn-SOD, and Cu/Zn-SOD, in the cortex of the left hemisphere was measured according to the respective reagent manufacturers' protocol and previously described methods [21].

### 2.5. Determination of ROS Levels

The cells were stained with 10 *μ*M dihydroethidium (DHE), an ROS probe, for 30 min in a dark humidified chamber. After being quickly washed with PBS, the cells were imaged using a fluorescence microscope and analyzed via NIH ImageJ software.

### 2.6. Hematoxylin and Eosin (HE) Staining and Immunofluorescence Staining

HE staining and immunofluorescence staining were performed according to a method that we have previously described [28]. For HE staining, the brain tissues were cut into coronal sections. Then, the sections were fixed, rinsed, and stained with hematoxylin and eosin, after which they were imaged with a light microscope.

For immunofluorescence staining, coronal sections of brain tissues were washed, blocked, and subsequently incubated overnight with rabbit anti-NeuN (1 : 200 dilution, ab177487, Abcam, USA) or mouse anti-IL-1*β* (1 : 100; sc-32294, Santa Cruz Biotechnology, USA). The following day, the sections were incubated with secondary antibodies and stained with a commercial TUNEL staining kit (Apo Alert DNA Fragmentation Assay kit; Clontech, BD Biosciences, Palo Alto, CA, USA) and diamidino-2-phenylindole (DAPI, 1 : 1000, C1002, Beyotime). The purpose of TUNEL staining is to show apoptotic cells, and the purpose of NeuN staining is to display neurons.

### 2.7. Reverse Transcription Real-Time Quantitative Polymerase Chain Reaction (RT–qPCR)

Total RNA from the injured cortex was isolated using a TRIzol kit (Invitrogen, Thermo Fisher Scientific, USA). RNA (2 *μ*g) was reverse transcribed to complementary DNA (cDNA) using the Reverse Transcription System (Invitrogen, Thermo Fisher Scientific) in accordance with the manufacturer's protocol. RT–PCR was carried out in a 20 *μ*L reaction system containing cDNA SYBR Green Master Mix (TaKaRa, Japan) and specific primers according to the manufacturer's instructions. The primers were designed for ATP5G3, PTGS2, CS, IREB2, and RPL8; the sequences of these primer pairs are listed in Table 1.

### 2.8. Transmission Electron Microscopy (TEM)

Rat brain tissues (2 × 2 mm) from the cortex of the lesioned side were fixed in phosphate-buffered glutaraldehyde (2.5%) and osmium tetroxide (1%), sliced into 50 nm-thick sections, stained with uranium acetate and lead citrate, and finally imaged with a transmission electron microscope (Hitachi HT7700, Japan).

### 2.9. Western Blot Analysis

The frozen cortex tissue and cell samples were washed twice with ice-cold phosphate buffered saline (PBS) and were completely lysed in ice-cold lysis buffer containing phenylmethanesulfonyl fluoride and phosphatase and protease inhibitors. The total cell lysates were centrifuged at 12,000 rpm for 20 min at 4°C. Afterward, the supernatant was collected to determine the quantity of protein in the samples with a Pierce BCA Protein Assay Kit. Equal amounts of proteins (10-20 mg) were isolated from the left cortex or primary cortical neurons, separated by SDS–PAGE (12%), and then electrotransferred onto polyvinylidene fluoride (PVDF) membranes. The membranes were blocked, incubated with rabbit anti-HMGB1 (1 : 1000, ab18256, Abcam, USA) and rabbit anti-GPX4 (1 : 1000; ab125066; Abcam, USA), washed, and incubated with secondary antibodies. The proteins were visualized by enhanced chemiluminescence (Pierce, Rockford, IL, USA, #32106) and quantified with NIH ImageJ software.

### 2.10. Statistical Analysis

Student's *t*-test was used to analyze the difference between two groups, and one-way ANOVA followed by multiple pairwise comparisons with the Bonferroni correction was used to evaluate the differences among more than two groups. A *P* value <0.05 was defined as statistically significant.

## 3. Results

### 3.1. Glycyrrhizin Reduced Cortical Neuronal Loss after HIBD

**Figure 1:**
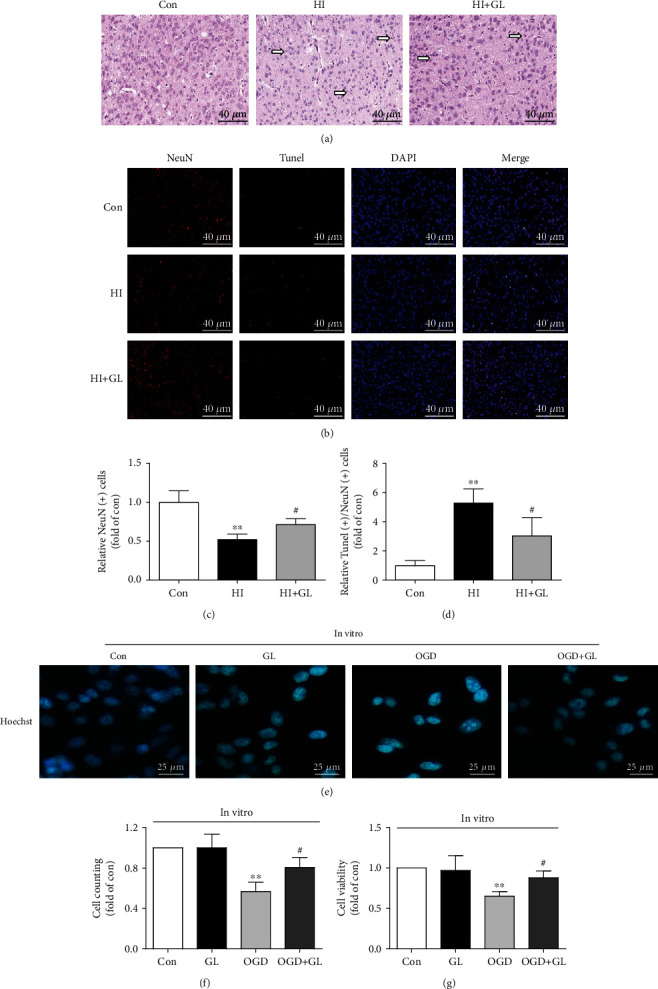
Glycyrrhizin improves cortical neuronal loss after HIBD and OGD. (a) HE-stained coronal sections from each group. The arrow indicates shrunken neuronal nuclei and sparse neurons. (b) Immunofluorescence labeling of TUNEL and NeuN in the cerebral cortex 72 h after HI (*n* = 6). (c) The statistical analysis of NeuN (+) cell counts (*n* = 6). (d) Statistical analysis of TUNEL (+)/NeuN (+) cell counts (*n* = 6). (e) Representative immunofluorescence of nuclear staining in primary cortical neurons. (f, g) Cell counting and cell viability in vitro (*n* = 4). ^∗∗^*P* < 0.01 vs. the Con group; #*P* < 0.05 vs. the HI group or OGD group.

First, at P10, we observed left cortex neuronal morphology by HE staining. Compared with the Con group, the HI group had sparse, disarranged neurons with shrunken nuclei, and these effects were reduced in the HI + GL group (Figure 1(a)). NeuN immunofluorescence labeling in conjunction with TUNEL staining was performed on neurons to detect neuronal damage (Figures 1(b)–1(d)). In the HI group, the number of NeuN-immunopositive cells in the cortex decreased significantly compared with the Con group, but the number of TUNEL/NeuN-double-positive cells increased significantly (Figures 1(b)–1(d)). Treatment with GL after HI induction increased the number of NeuN-positive neurons and reduced the number of TUNEL/NeuN-double-positive cells in the cortex area (Figures 1(b)–1(d)).

To further assess whether GL contributed to the cortical neuron damage induced by OGD at the cellular level in vitro, we assessed the number and viability of cortical neuron. Nuclear staining showed that the number of nuclei decreased in the OGD group and could be rescued in the OGD + GL group (Figure 1(e)). Similar protective effects of GL on OGD-induced cell damage were observed in terms of cell counts and cell viability (Figures 1(f) and 1(g)). These results suggest that inhibiting HMGB1 mitigates cortical neuronal damage after HIBD and OGD.

### 3.2. HMGB1 Depletion Rescued HI-Induced Neuronal Ferroptosis

**Figure 2:**
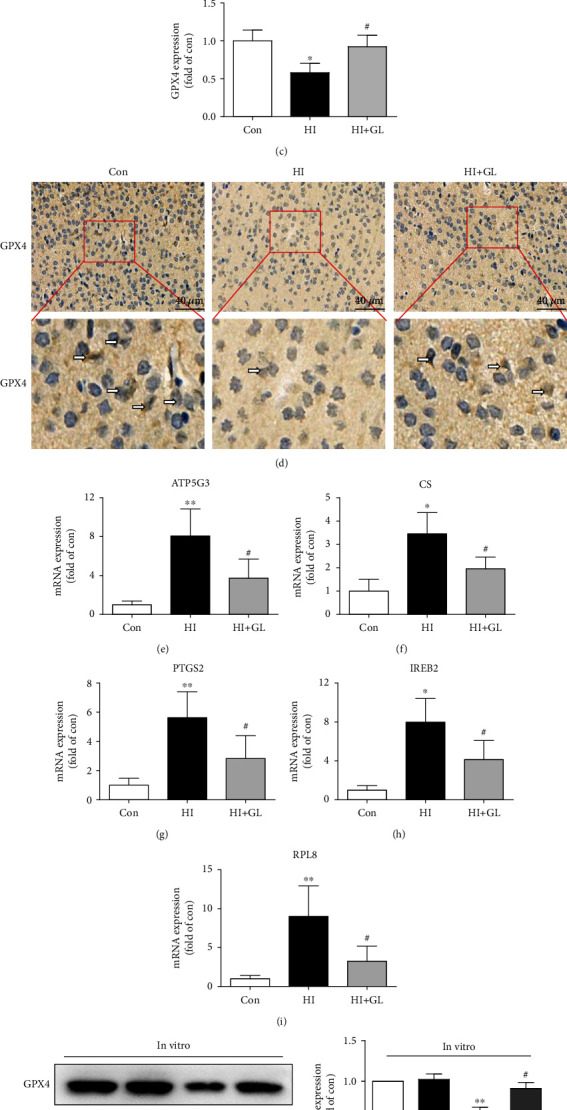
Glycyrrhizin inhibits HI-induced neuronal ferroptosis. (a)–(c) Western blots and average data for HMGB1 and GPX4 in the injured cortex at 72 h after HI (*n* = 5). (d) Representative immunohistochemical images of the GPX4 expression in the cerebral cortex. The arrows indicate GPX4+ cells. (e)–(i) Ferroptosis-related genes, including ATP5G3, CS, PTGS2, IREB2, and RPL8, were analyzed by qPCR (*n* = 5). (j) Western blots and the average data for GPX4 in vitro (*n* = 3). ^∗^*P* < 0.05, ^∗∗^*P* < 0.01 vs. the Con group; #*P* < 0.05 vs. the HI group or OGD group.

To determine whether GL could protect against neuronal damage after HIBD by inhibiting ferroptosis, we next assessed the protein levels of GPX4 and the mRNA levels of ferroptosis-related genes. Western blot analysis revealed that GL markedly suppressed the elevated expression of HMGB1 in HIBD (Figures 2(a) and 2(b)). However, the GPX4 protein expression was significantly reduced following HIBD and restored in the HI + GL group (Figures 2(a) and 2(c)). Similarly, immunohistochemistry verified that GPX4 protein levels were depleted in the HI group and increased following GL injection (Figure 2(d)). The mRNA levels of ferroptosis-related genes, including ATP5G3, CS, PTGS2, IREB2, and RPL8, were increased significantly in the HI group compared with the Con group and markedly reduced compared with the HI + GL group (Figures 2(e)–2(i)). GPX4 protein levels were decreased in the OGD group and improved following GL treatment in vitro (Figure 2(j)). These results show that GL can inhibit not only the expression of HMGB1 but also the occurrence of neuronal ferroptosis in HIBD.

### 3.3. Glycyrrhizin Ameliorated Mitochondrial Injury and Oxidative Stress Levels in the Cortex following HIBD

**Figure 3:**
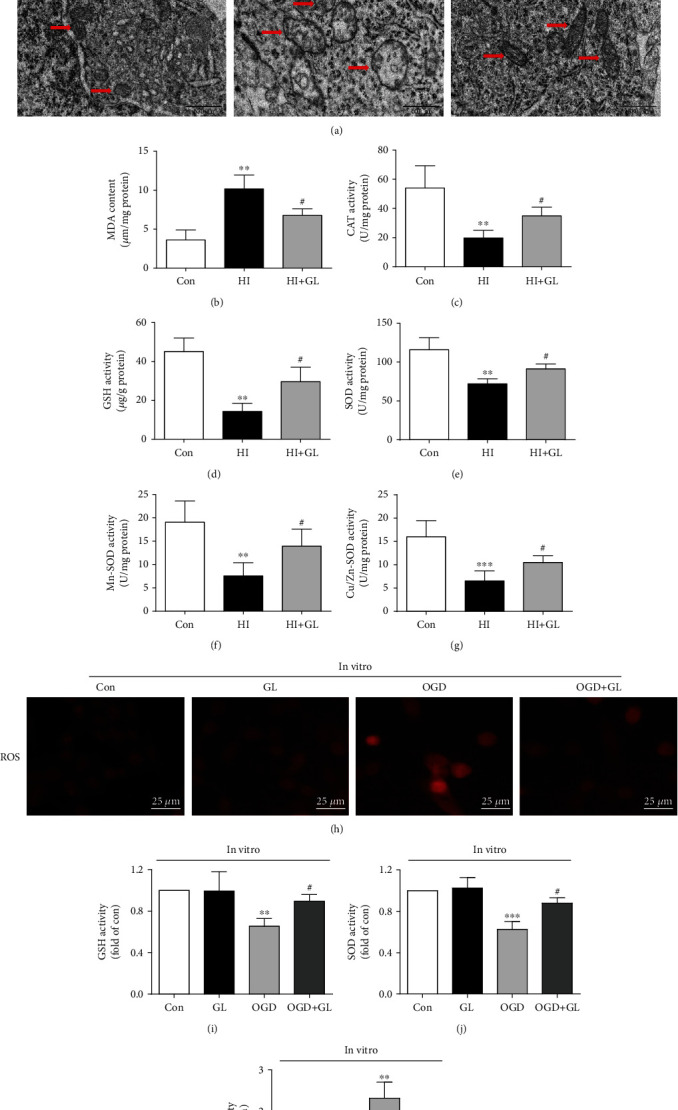
Glycyrrhizin ameliorates HIBD-induced mitochondrial injury and oxidative stress. (a) Representative TEM images of neurons and mitochondria in neurons in the injured cortex. (b)–(g) The level of MDA and the activity levels of CAT, GSH, SOD Mn-SOD, and Cu/Zn-SOD in different groups (*n* = 6). (h) Representative images of DHE (red) staining in cortical neuronal cells. (i)–(k) The levels of GSH, SOD, and MDA in vitro (*n* = 4). ^∗∗^*P* < 0.01, ^∗∗∗^*P* < 0.001 vs. the Con group; #*P* < 0.05 vs. the HI group or OGD group.

To further confirm the occurrence of ferroptosis, we observed the changes in neuronal ultrastructure via TEM. In contrast to the normal mitochondria in the Con group, morphologically abnormal mitochondria were observed in the HI group; these abnormalities were closely associated with ferroptosis, including smaller mitochondria, the disappearance of mitochondrial cristae, mitochondrial swelling, and mitochondrial vacuoles (Figure 3(a)). However, in the HI + GL group, the number of abnormal mitochondria was significantly reduced, and the morphology of abnormal mitochondria was improved (Figure 3(a)). To investigate whether GL protects against neuronal ferroptosis by adjusting the redox balance, the abundance of oxidants and antioxidants was measured in rat cortical tissue. The increase in the MDA level in the HI group was markedly inhibited in the HI + GL group (Figure 3(b)), indicating that tissue oxidative lipid damage was reduced. The activity levels of antioxidative enzymes, including CAT, GSH, SOD, Mn-SOD, and Cu/Zn-SOD, were reduced in the HI group in comparison with the Con group, and these changes were significantly reversed in the HI + GL group (Figures 3(c)–3(g)). Additionally, we measured ROS levels in vitro using DHE. Interestingly, the increase in red fluorescence in the OGD group was clearly mitigated in the OGD + GL group (Figure 3(h)). Furthermore, the results of GSH, SOD, and MDA detection in vitro were similar to the results in vivo (Figures 3(i)–3(k)). Therefore, it is concluded that mitochondrial damage and redox imbalance induced by HIBD or OGD can be rescued with GL; these mechanisms might play a key role in the therapeutic effects of GL on HIBD-induced neuronal ferroptosis.

### 3.4. Glycyrrhizin Prevents Neuroinflammation after HIBD

**Figure 4:**
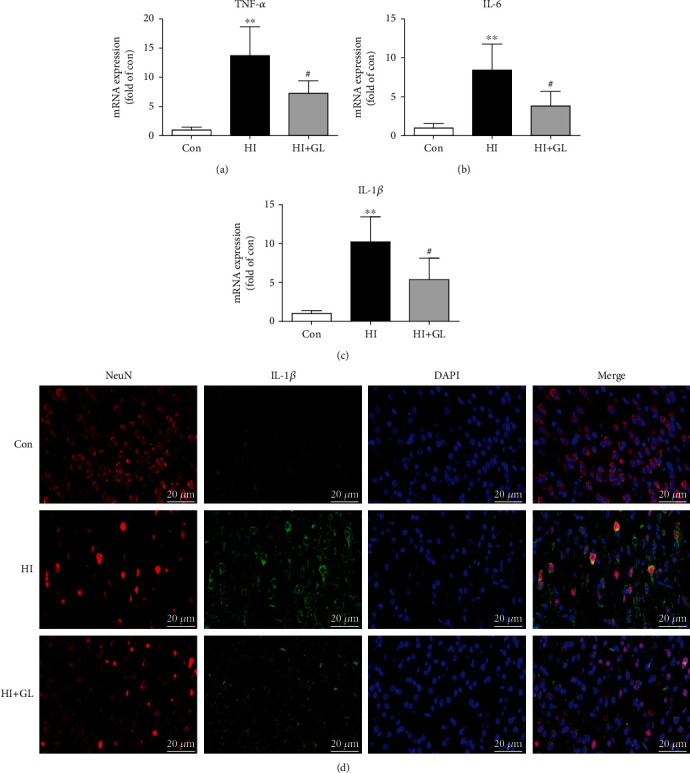
Inhibiting HMGB1 alleviates the activation of neuroinflammation. (a)-(c) The proinflammatory cytokines in the injured cortex at 72 h after HI, including TNF-*α*, IL-6, and IL-1*β*, were analyzed by qPCR (*n* = 5). (d) Representative immunofluorescence images of the IL-1*β* expression in cortical neurons. ^∗∗^*P* < 0.01 vs. the Con group; #*P* < 0.05 vs. the HI group.

To further test the role of GL in HIBD, the relevant inflammatory factors TNF-*α*, IL-6, and IL-1*β* were detected by qRT–PCR. The results indicated that the levels of TNF-*α*, IL-6, and IL-1*β* were significantly increased at 72 h following HIBD in the injured cortex, which was obviously suppressed by GL treatment (Figures 4(a)–4(c)). Furthermore, the immunofluorescence of the IL-1*β* expression in injured cortical neurons further suggested that there was a significant decrease in the expression of IL-1*β* in the HI + GL group compared with the HI group (Figure 4(d)).

### 3.5. Glycyrrhizin Improves Neuronal Ferroptosis through the GPX4 Axis

**Figure 5:**
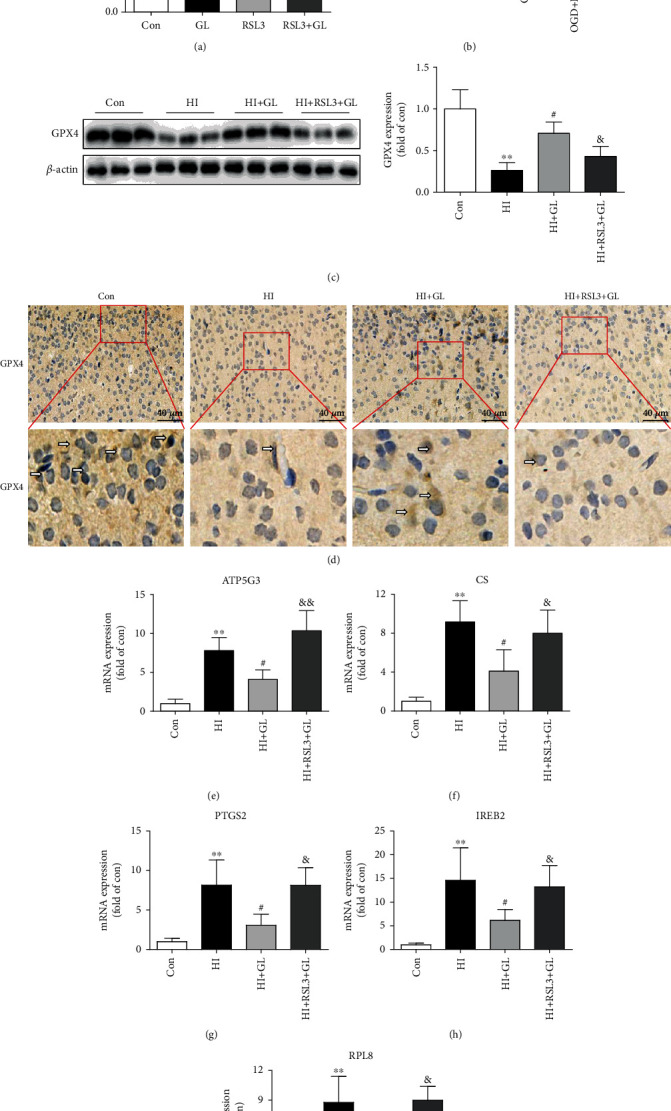
GPX4 inhibition with RSL3 blocked the glycyrrhizin-induced improvement in neuronal ferroptosis following HIBD. (a, b) Western blots and the average data for GPX4 in vitro (*n* = 3). (c) Representative Western blots and the average data of GPX4 in the different in vivo experiments (*n* = 6). (d) Representative immunohistochemical images of the GPX4 expression in the injured cortex at 72 h after HI. (e)–(i) Ferroptosis-related genes, including ATP5G3, CS, PTGS2, IREB2, and RPL8, were analyzed by qPCR (*n* = 6). ^∗∗^*P* < 0.01 vs. the Con group; #*P* < 0.05 vs. the HI, RSL3 or OGD group; &*P* < 0.05, &&*P* < 0.01 vs. the HI + GL or OGD + GL group.

To further verify that GL reduces HIBD-induced neuronal damage by inhibiting neuronal ferroptosis, RSL3, a ferroptosis inducer and an inactivator of GPX4, was used in the in vivo and in vitro experiments. Interestingly, we found that RSL3-induced downregulation of GPX4 in vitro could be rescued by GL in the RSL3 + GL group (Figure 5(a)). Furthermore, OGD-induced GPX4 inhibition was improved in the OGD + GL group, and this improvement was attenuated by RSL3 in the OGD + GL + RSL3 group (Figure 5(b)). In vivo, we found that GL treatment restored the GPX4 level to normal after HIBD, while treatment with RSL3 reversed this effect (Figure 5(c)). Consistently, compared with the HI + GL group, the expression of GPX4 in immunohistochemistry of the injured cortex was suppressed by RSL3 in the HI + GL + RSL3 group (Figure 5(d)). Additionally, the RT–qPCR results revealed that compared with the HI + GL group, the mRNA levels of ATP5G3, CS, PTGS2, IREB2, and RPL8 were significantly increased in the HI + GL + RSL3 group (Figures 5(e)–5(i)). These findings suggest that inhibiting HMGB1 alleviates neuronal ferroptosis via the GPX4 signaling pathway.

### 3.6. RSL3 Impedes GL to Ameliorate Neuroinflammation in HIBD

**Figure 6:**
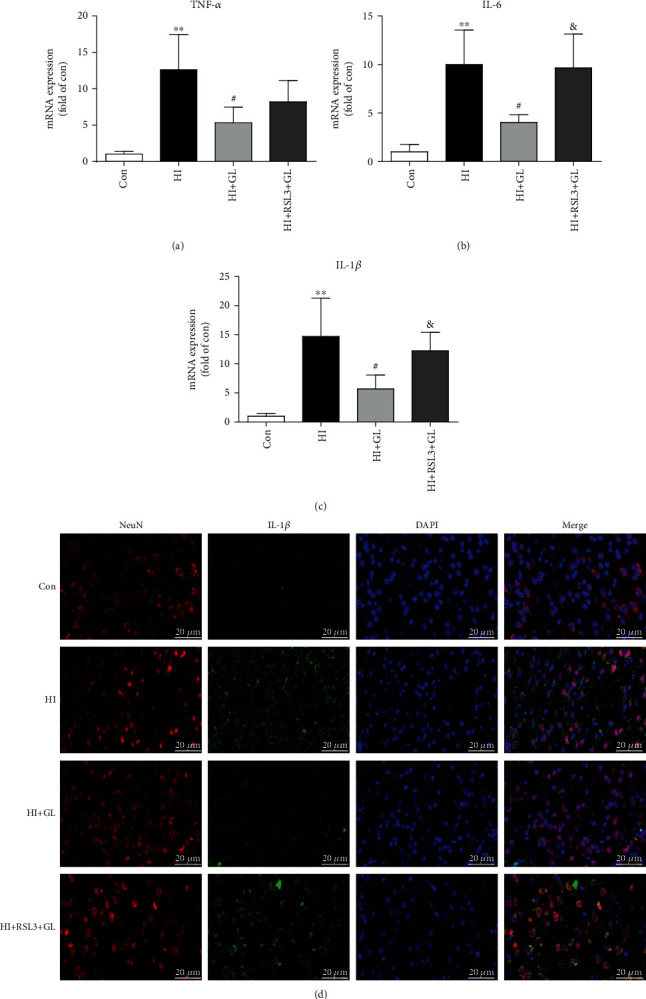
RSL3 hinders glycyrrhizin in ameliorating the activation of neuroinflammation. (a)–(c) The expression of TNF-*α*, IL-6, and IL-1*β* was analyzed by qPCR (*n* = 6). (d) Representative immunofluorescence images of IL-1*β* and NeuN staining. ^∗∗^*P* < 0.01 vs. the Con group; #*P* < 0.05 vs. the HI group; &*P* < 0.05 vs. the HI + GL group.

Subsequently, we test whether GL exerts a protective effect by suppressing ferroptosis-induced inflammation. There was no significant difference in TNF-*α* levels between the HI + GL group and the HI + GL + RSL3 group (Figure 6(a)). Compared with the HI + GL group, however, the HI + GL + RSL3 group had a significant increase in the levels of IL-6 and IL-1*β* in the injured cortex (Figures 6(b) and 6(c)). Moreover, the ferroptosis-related inflammatory product IL-1*β* was also indicated by the immunofluorescence results, which showed that IL-1*β* was more highly expressed in the cortical neurons of the HI + GL + RSL3 group than in those of the HI + GL group (Figure 6(d)).

### 3.7. Glycyrrhizin Improves Cortical Neuronal Loss Related to Ferroptosis through the GPX4 Axis

**Figure 7:**
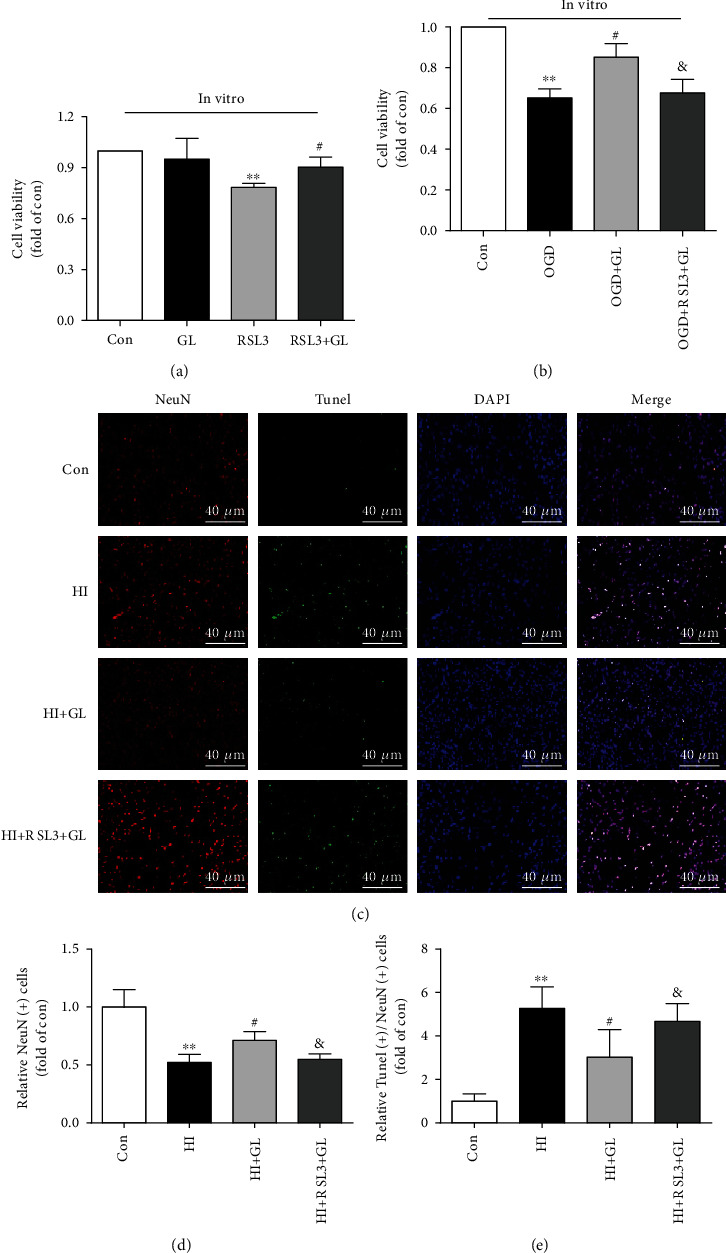
HIBD-induced cortical neuronal loss can be alleviated by glycyrrhizin via the GPX4 axis. (a) The cell death induced by RSL3 was evaluated using the MTT assay (*n* = 4). (b) GL increased the viability of cortical neurons subjected to OGD (*n* = 4). (c) Representative immunofluorescent images of TUNEL and NeuN staining in the injured cortex at 72 h after HI (*n* = 6). (d) The statistical analysis of NeuN (+) cell counts (*n* = 6). (e) The average data for TUNEL (+)/NeuN (+) cell counts (*n* = 6). ^∗∗^*P* < 0.01 vs. the Con group; #*P* < 0.05 vs. the HI, RSL3 or OGD group; &*P* < 0.05 vs. the HI + GL or OGD + GL group.

To further investigate the protective effect of GL on OGD-induced cell death via ferroptosis, we assessed cell viability by MTT in vitro. First, RSL3 significantly reduced cell viability in vitro, and GL ameliorated RSL3-induced cytotoxicity (Figure 7(a)). Furthermore, GL treatment improved cell viability following OGD, while treatment with RSL3 reversed this effect (Figure 5(b)). In addition, TUNEL staining and immunohistochemical neuronal staining were jointly performed to further verify that GL rescued HIBD-induced neuronal damage through ferroptosis related to the GPX4 axis (Figure 7(c)). The number of NeuN-immunopositive cells in the cortex was significantly decreased in the HI + GL + RSL3 group compared with the HI + GL group (Figures 7(c) and 7(d)). However, there were more TUNEL/NeuN-double-positive cells in the HI + GL + RSL3 group than in the HI + GL group (Figures 7(c) and 7(e)). Therefore, these results indicate that GL downregulates the level of neuronal ferroptosis by promoting the GPX4 expression, and that this effect of GL can improve cortical neuronal loss in HIBD and OGD.

## 4. Discussion

With limited treatment options, HIBD remains an imposing clinical problem and is particularly closely related to the quality of life of newborns [29]. Previous studies have demonstrated that the exhaustion of brain cell energy production, oxidative stress injury, mitochondrial damage, and inflammation leads to the occurrence and development of cell damage in HIBD [26, 29–31]. However, the specific mechanism of neuronal damage in HIBD is unclear.

HMGB1, a damage-associated molecule, can stimulate and amplify inflammatory responses and ultimately induce the loss of neurons in HIBD [30]. In previous studies, HMGB1 was released from cells beginning 6 h after HI; this molecule is considered a sensitive marker of HIBD in neonates [24]. Recent studies have shown that HMGB1 inhibition can reduce infarct size and ameliorate neurobehavioral impairments in neonatal HIBD, suggesting that GL can be used as a neuroprotective agent for the treatment of HIBD [26, 30]. In our study, we further observed that inhibiting HMGB1 with GL could significantly reduce the amount of neuronal death and ameliorate cortical neuronal loss in HIBD by suppressing ferroptosis.

Ferroptosis is a recently discovered form of necrotic cell death that is characterized by the iron-dependent accumulation of ROS [32]. Dysregulation of ferroptosis is closely associated with various diseases, including cancer, ischemia–reperfusion injury, Parkinson's disease, stroke, and neurodegeneration [32, 33]. Our recent research has also shown an intimate link between HIBD and ferroptosis [15]. The characteristics of ferroptosis are iron-dependent ROS accumulation, inflammation, and mitochondria-dependent damage within the cell, which are also the underlying pathogeneses of HIBD [4, 5, 20, 34, 35]. In the current study, we investigated the effect of GL on oxidative stress levels, mitochondrial damage, and neuroinflammation in HIBD. The results indicated that these injuries, including increased MDA levels; decreased CAT, GSH, SOD, Mn-SOD, and Cu/Zn-SOD levels; increased expression of ferroptosis-related genes, including ATP5G3, CS, PTGS2, IREB2, and RPL8; and induced mitochondrial damage and neuroinflammation in the HIBD rat model, could be alleviated by GL administration; these effects were accompanied by a decrease in neuronal ferroptosis. These findings further show that GL may play a neuroprotective role in HIBD as a ferroptosis inhibitor.

Ferroptosis inhibitors, including ferrostatin-1 and liproxstatin-1, have been demonstrated to have a protective effect in the transient middle cerebral artery occlusion model of ischemic stroke in mice and rats and in intracerebral hemorrhage in mice [18, 21]. Owing to the difficulty of delivering these two ferroptosis inhibitors across the blood–brain barrier, their clinical applications to CNS diseases are limited [18, 21]. GL, a major constituent of licorice root, has been demonstrated to pass through the blood–brain barrier and exert a neuroprotective effect when administered by intraperitoneal injection [22]. The protective effect of GL against mitochondrial injury and oxidative stress has also been confirmed in pathological conditions [36]. GL may be associated with the improvement of mitochondrial electron transfer chain activity, transmembrane potential, and decreased aconitase activity in the tricarboxylic acid cycle, which, in turn, decreases the formation of ROS and results in the improvement of mitochondrial function [36–38]. The present study demonstrated that GL not only inhibited ferroptosis induced by RSL3 and OGD in vitro but also inhibited ferroptosis induced by HIBD in vivo. More importantly, GL may improve oxidative stress imbalance and mitochondrial damage, alleviate their downstream inflammatory factors, and ultimately reduce ferroptosis and damage to cortical neurons following HIBD by regulating the expression of HMGB1 and GPX4. Therefore, these findings provide conclusive evidence that GL may inhibit RSL3- and HIBD-induced ferroptosis and reveal the potential of GL to treat diseases that are characterized by ferroptosis, including HIBD.

To date, research has shown that ferroptosis can be controlled by GPX4 and radical-trapping antioxidants [39]. GPX4 is an essential regulator that scavenges lipid peroxides under oxidative stress [40]. Reduced GSH is exploited by the antioxidant enzyme GPX4 to convert phospholipid hydroperoxides to lipid alcohols [33]. Inactivation of GPX4 promotes excessive lipid peroxide formation and results in mitochondrial damage, which plays an important role in the occurrence of ferroptosis [9, 41]. RSL3 drives ferroptosis by binding and inactivating GPX4 [42]. To elucidate the function and mechanism of HMGB1 and ferroptosis, we further analyzed the effects of RSL3 and GL on the activation of GPX4 and neuronal damage. We found that the expression of GPX4 was increased by GL in HIBD; however, this effect did not occur in the presence of RSL3 in rats subjected to HI. Furthermore, GPX4 inhibition using RSL3 eliminated GL-mediated amelioration of neuronal damage, improvement of neuronal ferroptosis, and reduction of neuroinflammation in HIBD rats. Taken together, these findings further demonstrate that GL improves neuronal loss, ameliorates mitochondrial damage, and reduces neuroinflammation following HIBD by inhibiting neuronal ferroptosis through the HMGB1/GPX4 pathway.

One limitation of our study is that we showed only the connection between HMGB1 and neuronal ferroptosis; the other modes of neuronal death, including apoptosis, autophagy, and pyroptosis, were not examined here. Another possible limitation is that this experiment focused on the acute phase of HIBD (72 h) and did not observe the long-term effect. GL is considered an HMGB1 inhibitor and can also inhibit superoxide, NO, and ONOO- production, helping to reduce HMGB1 and confer neuroprotection in ischemic brain injury [43]. Our research confirmed that GL can suppress MDA levels and increase the levels of CAT, GSH, SOD, Mn-SOD, and Cu/Zn-SOD, which implies that the neuroprotective effects of GL in reducing ferroptosis could also be attributable to its antioxidant activity in HIBD. Therefore, the antioxidant activity and inhibition of HMGB1 are both involved in the suppression of ferroptosis by GL in HIBD. The functions of antioxidant activity and HMGB1 inhibition may simultaneously exist. However, in this study, we did not further investigate whether antioxidant activity or HMGB1 inhibition plays a more important role in the regulation of ferroptosis by GL in HIBD. Finally, this study merely indicated the effect of HMGB1 inhibition on neuronal ferroptosis by pharmacological approaches and perspectives and did not explore the deeper mechanisms apart from the GPX4 signaling pathway. Therefore, these limitations need to be further addressed in subsequent research.

## 5. Conclusion

In summary, we indicated for the first time that GL could suppress the occurrence of neuronal ferroptosis, inhibit oxidative stress, reduce mitochondrial damage, and ameliorate neuroinflammation in HIBD via the HMGB1/GPX4 pathway. Collectively, these findings highlight the potential of HMGB1 signaling antagonists to treat neuronal damage by suppressing ferroptosis, provide new and unique insights into GL as a neuroprotective agent and a ferroptosis inhibitor, and suggest new prevention and treatment strategies for diseases featuring ferroptosis, especially HIBD.

## Figures and Tables

**Table 1:** Primer sets used for qPCR.

	Forward primer (5′-3′)	Reverse primer (5′-3′)
ATP5G3	GACTAGGACTGGAGAGGGCT	ATACCAGCACCAGAACCAGC
PTGS2	CTTCGGGAGCACAACAGAGT	TTCAGAGGCAATGCGGTTCT
IREB2	GGGAATTCTTGGGTGGGGAG	AACAAACTTTCCAGCCACGC
CS	GCTACAGAAGGAAGTCGGCA	CCCGAGTTGAGTGTGTTCCA
RPL8	GCAAGCCTTCCACTATCCGA	CAATGAGACCCACTTTGCGC
TNF-*α*	GCATGATCCGAGATGTGGAACTGG	CGCCACGAGCAGGAATGAGAAG
IL-6	AGGAGTGGCTAAGGACCAAGACC	TGCCGAGTAGACCTCATAGTGACC
IL-1*β*	ATCTCACAGCATCTCGACAAG	CACACTAGCAGGTCGTCATCC
*β*-Actin	CACGATGGAGGGGCCGGACTCATC	TAAAGACCTCTATGCCAACACAGT

## Data Availability

The data that support the findings of this study are available from the corresponding author upon reasonable request.
